# Polyploid Giant Cancer Cells, a Hallmark of Oncoviruses and a New Therapeutic Challenge

**DOI:** 10.3389/fonc.2020.567116

**Published:** 2020-10-14

**Authors:** Georges Herbein, Zeina Nehme

**Affiliations:** ^1^Pathogens & Inflammation/EPILAB Laboratory, EA 4266, University of Franche-Comté, Université Bourgogne Franche-Comté (UBFC), Besançon, France; ^2^Department of Virology, CHRU Besancon, Besançon, France; ^3^Faculty of Sciences, Lebanese University, Beirut, Lebanon

**Keywords:** PGCC, oncoviruses, tumor heterogeneity, chemotherapy resistance, radiotherapy resistance

## Abstract

Tumors are renowned as intricate systems that harbor heterogeneous cancer cells with distinctly diverse molecular signatures, sizes and genomic contents. Among those various genomic clonal populations within the complex tumoral architecture are the polyploid giant cancer cells (PGCC). Although described for over a century, PGCC are increasingly being recognized for their prominent role in tumorigenesis, metastasis, therapy resistance and tumor repopulation after therapy. A shared characteristic among all tumors triggered by oncoviruses is the presence of polyploidy. Those include Human Papillomaviruses (HPV), Epstein Barr Virus (EBV), Hepatitis B and C viruses (HBV and HCV, respectively), Human T-cell lymphotropic virus-1 (HTLV-1), Kaposi's sarcoma herpesvirus (KSHV) and Merkel polyomavirus (MCPyV). Distinct viral proteins, for instance Tax for HTLV-1 or HBx for HBV have demonstrated their etiologic role in favoring the appearance of PGCC. Different intriguing biological mechanisms employed by oncogenic viruses, in addition to viruses with high oncogenic potential such as human cytomegalovirus, could support the generation of PGCC, including induction of endoreplication, inactivation of tumor suppressors, development of hypoxia, activation of cellular senescence and others. Interestingly, chemoresistance and radioresistance have been reported in the context of oncovirus-induced cancers, for example KSHV and EBV-associated lymphomas and high-risk HPV-related cervical cancer. This points toward a potential linkage between the previously mentioned players and highlights PGCC as keystone cancer cells in virally-induced tumors. Subsequently, although new therapeutic approaches are actively needed to fight PGCC, attention should also be drawn to reveal the relationship between PGCC and oncoviruses, with the ultimate goal of establishing effective therapeutic platforms for treatment of virus-associated cancers. This review discusses the presence of PGCCs in tumors induced by oncoviruses, biological mechanisms potentially favoring their appearance, as well as their consequent implication at the clinical and therapeutic level.

## Introduction

Tumors are perceived as composite systems that diversify at the molecular, cellular, and architectural levels, accounting for phenotypic and functional heterogeneity (1). With divergence at the genetic, epigenetic, transcriptomic, proteomic, and cellular levels tumor heterogeneity is regarded as an essential barrier hindering the development of curative anti-cancer therapies (2). In this perspective, giant multinucleated or large nucleated cells denoted as polyploid giant cancer cells (PGCC) appear to significantly contribute to the shaping and the composition of cancer genomes and tumor evolution, rendering them crucial therapeutic targets to fight therapy resistance (3). This paradigm establishes a new conceptual framework and relocates the attention on the centuries-old embryological theory of cancer beside the somatic mutation and the clonal selection theories (4). In this re-emerging consensus, it is speculated that PGCC act as keystone cancer sub-population and actuators of an endogenous mechanism of somatic cell de-differentiation or reprogramming, which set the stage for cancer initiation, therapeutic resistance and relapse in metastatic disease (5). This genomic reorganization is conceptualized by Niu et al. as a “giant cell cycle” depicted in four distinct but overlapping phases designed as initiation, self-renewal, termination, and stability, where a process of reductive division known as depolyploidization results in the formation of diploid progeny cells with novel or altered genotypes (6).

On the other hand, viral oncogenesis is a multistep process that rely on complex molecular mechanisms and multiplex interplay between the host and oncogenic viruses (7). The latter account for ~12% of human cancers and includes Human Papillomaviruses (HPV), Epstein Barr Virus (EBV), Hepatitis B and C viruses (HBV and HCV, respectively), Human T-cell lymphotropic virus-1 (HTLV-1), Kaposi's sarcoma herpesvirus (KSHV) and Merkel polyomavirus (MCPyV) (8). Although those pathogens hijack the cellular machinery to establish infection, replication and persistence, as well as to promote tumorigenesis through common pathways, targeted therapies and definitive curative clinical interventions are still lacking (9). This is due not only to limitation of equivalent animal models, but also due to the enigma associated with some mechanistic aspects of cancer induction by infectious agents and their disparate nature (10). In this context, dissecting the clonal sub-population of those tumors at the genetic level appears to be an essential clue to understand and decipher the aspects of cancer initiation and progression and the subsequent extrapolation toward prognosis and treatment. Genuinely, genomic instability, in the form of aneuploidy and most importantly polypoidy appear to be a shared characteristic among oncogenic viruses, where distinct viral proteins can pave the road, through various mechanisms, for the initial trigger of cellular transformation (11).

As a thorough causality between oncoviruses, polyploidy, and tumorigenesis has not yet been established, this review will discuss the presence or alternatively the induction of a polyploid giant cancer cell phenotype in the context of viral infections, as well as the molecular pathways that might mediated or favor this mechanism and its clinical implication in terms of therapy resistance.

### Polyploidy and Oncoviruses

Although oncogenic viruses converge in common features from a broad perspective, divergent genomes, oncogenic factors, cellular tropism, and disease prevalence distinguish each of those infectious agents (12, 13). Nonetheless, polyploidy appears to be a crossroad and a biological equivalency trait shared among oncoviruses, where the latter could be regarded as an initiating engine that fuels phenotypic diversity. Such heterogeneity, characterized partly by the presence of proportion of giant cells with a highly enlarged nucleus or multiple nuclei in *in vitro* or *in vivo* systems is discussed in following section.

#### Human Papillomavirus (HPV)

Belonging to *Papillomaviridae* family, Human papillomavirus (HPV) is a small, double-stranded, circular DNA virus with a non-enveloped icosahedral capsid (14). Encompassing more than 200 types, HPVs have proven their tropism for cutaneous and mucosal epithelia (15). Besides, and based on their relative malignancy, HPVs are clustered into two types: low-risk (LR) HPV and high-risk (HR) HPV, where the former is linked to low-grade lesions as benign warts and the latter is interrelated to high-grade cervical lesions and cancers (16). Although HR HPV are further divided into 20 types (17), HPV-16 and−18 are the most studied ones as they account for 62.6 and 15.7% of cervical cancer cases respectively, with a special highlight on the two HPV-encoded oncoproteins E6 and E7 (18, 19). In this context, the detection of polyploidy is finely described. Indeed, tetraploidy is identified as an early and common event during cervical carcinogenesis (20). Further, an exceedingly significant correlation is established between HR-HPV infection, the presence and/or the development of a high-grade squamous intraepithelial lesion (HGSIL) and the detection of polyploidy (21, 22). It has been shown that infection of squamous intraepithelial lesions of the cervix with high-risk but not low-risk HPVs results in basal keratinocyte tetrasomy (23, 24). Expression of both E6 and E7 oncoproteins resulted in higher percentage of cells with >4n DNA content in neonate foreskin keratinocytes compared to control cells (25). In addition, spontaneous polyploidization was detected in HPV-16 E6-expressing fibroblasts at late passages (26), as well as in primary human keratinocytes (PHK) expressing E6 (27) and in the immortalized retinal pigment epithelial cells RPE1 in the presence of p53 through a p53-independent mechanism of E6-induced polyploidy (28). Not restricted to E6, HPV-16 E7 induces polyploidy formation in response to DNA damage (29) with the cell division cycle 6 (cdc6) protein as an important mediator (30). Polyploidization was also detected in human keratinocyte cell lines PHK16-I, PHK16-II, PHK16-L1, and PHK16-L2 and in primary mouse keratinocytes upon HPV-16 E7 expression (31), as well as in RPE1 cells expressing E7 (32). Interestingly, the expression of HPV-16 E5, a third viral protein with transforming potential upon independent expression (33), resulted not only in enlarged nuclei, but also in an increase in cellular DNA content and chromosomal number (34). Lastly, HPV-18 E7 transduction resulted in the emergence of cells with enlarged nuclei or alternatively binucleated or trinucleated cells in cultures of differentiated keratinocytes (35). Thus, the expression of HPV oncogenes could induce polyploidy as an early event during cervical carcinogenesis.

#### Epstein–Barr Virus (EBV)

Epstein–Barr virus (EPV) is a linear, double-stranded DNA virus classified in the family Herpesviridae, subfamily Gammaherpesvirinae (36). Although EBV can infect T lymphocytes or epithelial cells, it is well-thought-out to be a B-lymphotropic virus, where it establishes latency and induces proliferation of B lymphocytes (37). Based on the cell types for which EBV exhibits tropism, the virus is associated with a wide array of malignancies including several subset of lymphomas, for instance Hodgkin's lymphoma, diffuse large B-cell and Burkitt's lymphoma (BL), and a subset of carcinomas including gastric and nasopharyngeal carcinoma (NPC) (38). Genuinely, polyploidy is described in tissue biopsies of NPC where polynuclear giant cancer cells were detected, surrounded by an indefinite small nucleus-containing bodies, indicative of budding cells (39). In addition, increased volume and pronounced multinucleation with cells counting more than 12 nuclei were reported in a nasal mucosal neoplasm infected with EBV (40). Furthermore, EBV replication in epithelial NPC hybrid cells induced the formation of multinucleated giant cells (41). B cell infection with an EBV strain isolated from a nasopharyngeal carcinoma, predisposed to polyploidy with cells displaying several micronuclei or containing a single large polyploid nucleus (42). Besides, chromosomal integration of the viral genomic DNA into primary human B cells was associated with polyploidy in the generated lymphoblastoid cell lines (43). More specifically, expression of Epstein–Barr virus nuclear antigen 2 (EBNA2), a viral nuclear protein involved in EBV latency regulation and essential for B-cells immortalization (44) can induce micronuclei and multinucleated cell formation in the human laryngeal carcinoma HEp-2 and osteosarcoma U2-OSn cells (45). In line with this, transfection with EBNA3C, another viral latent protein crucial for B cells immortalization and with oncogenic property in primary rodent fibroblasts in co-operation with activated RAS (46), produced bi- and multi-nucleated cells in NIH3T3 fibroblasts and U2OS cells where distinct peaks corresponding to 6n and 8n and cells enclosing from 1 to 7 nuclei were detected (47). Lastly, stable expression of the latent membrane protein 1 (LMP1), a viral oncoprotein that provides essential survival signals (48) was associated with multinuclearity in a Burkitt's lymphoma cell line (49). This indicates that a close correlation between polyploidy and EBV infection or EBV-latent oncoproteins expression does exist.

#### Kaposi's Sarcoma-Associated Herpes Virus (KSHV)

Kaposi's sarcoma-associated herpes virus (KSHV) or human herpesvirus 8 (HHV-8) is a double stranded DNA gamma-2 herpesvirus (50). KSHV exhibits tropism toward a wide spectrum of cells including B cells, endothelial and epithelial cells, fibroblasts, keratinocytes, and dendritic cells (51). KSHV is identified as the etiological agent behind a heterogeneous group of malignancies, mainly Kaposi sarcoma (KS), a low-grade angiogenic vascular spindle cancer of endothelial cells and primary effusion lymphoma, in addition to multicentric Castleman disease, a non-cancer rare lymphoproliferative disease that can advance to plasmablastic lymphoma (52). In regards to polyploidy, KSHV infection of human umbilical vein endothelial cells (HUVECs), a model that mimics KSHV infection in KS tumor cells, induced a multinucleation state, associated with nuclei enlargement and shape irregularity (53). Activation of Rac1, a small GTPase suggested to be a key determinant of Kaposi sarcoma when triggered by host and viral genes, resulted in significant polyploidy in FVB/N transgenic mouse lines (54). On the other hand, the latency-associated nuclear antigen (LANA) is a multifunctional protein essential for efficient viral DNA replication and maintenance in latently infected tumor cells with a potential role in promoting oncogenesis (55). Constitutive expression of LANA in HeLa cells, a human cervical cancer cell line, in BJAB cells, a B cell line and in Rat1 fibroblasts induced a dramatic increase in the multinucleated phenotypes, characterized by cells with two or more polarized nuclei (56, 57). In addition, a KSHV cyclin D homolog denoted as K cyclin is recognized as a potential oncoprotein that interacts with cyclin-dependent kinases (CDKs) (58), phosphorylates a wide spectrum of substrates through complexing with CDK6 including p27 to trigger its degradation (59) and initiates DNA replication as well as cell cycle progression (60). Interestingly, K cyclin expression in mouse embryo fibroblasts yielded cells with enlarged nuclei as well as cells with multiple large multilobular ones (61) pointing toward a robust association linking KSHV to polyploidy.

#### Human T Cell Lymphotropic Virus (HTLV-1)

Acknowledged as the first human retrovirus to be discovered, human T cell lymphotropic virus (or T-cell leukemia virus) type 1 (HTLV-1) is a delta retrovirus belonging to *Retroviridae* family, subfamily *Orthoretrovirinae* (62). Although HTLV-1 is detected in multiple cell types, counting hematopoietic cells (e.g., monocytes, macrophages, dendritic cells) in addition to endothelial, synovial and glial cells, the virus preferentially infect CD4^+^ memory T cells, with both CD4^+^ and CD8^+^ T identified as viral reservoirs (63). In terms of induced malignancies, HTLV-1 is identified as the etiological agent behind adult T-cell leukemia/lymphoma (ATL), an aggressive lymphoproliferative non-Hodgkin's peripheral T-cell malignancy (64). In this context, HTLV-1 infection induces giant lymphoma cells with highly convoluted cerebriform nuclei (65). Distinctively, HTLV-1-encoded Tax is a pleiotropic oncoprotein indispensable for productive viral replication and direct or indirect activation of a variety of transcription pathways, along with T cells immortalization *in vitro* and transformation of rodent fibroblasts (66). Genuinely, Tax expression frequently prompts the generation of multinucleated polyploid cells (67). For instance, multinucleated giant cells with nuclei described as double, greatly enlarged, and/or highly lobulated were detected in mammalian cells highly expressing Tax (68, 69). Moreover, Tax-expressing HeLa cells exhibited enlarged cell sizes with greater than G2 DNA content compared to untransduced controls (70), with multinucleated cells showing giant lobulated nuclei (71). Likewise, HTLV-1 infected CD4^+^ clones displayed enlarged well-separated nuclei within binucleated and multinucleated cells, a morphological finding positively correlated with the degree of Tax expression (72). On the other hand, HTLV-1 accessory protein p30^II^, a multifunctional latency-maintenance factor, is also suggested to induce polyploidy in cooperation with c-Myc oncoprotein (73), although transfection of infectious HTLV-1 ACH. p30^II^ mutant provirus in HT0180 clones was able to induce multinucleation (74). Being a pleiotropic sensor, c-Myc oncogene is considered as master regulator and mediator of multiple cellular signals and transcriptional responses (75). c-Myc was shown to promote S-phase cell cycle entry (76), endomitosis and polyploidy formation (77), in addition to neoplastic cellular transformation (78). HTLV-1 p30^II^ distinctly enhances c-Myc transforming activity, engendering S-phase progression and polyploidy through the interaction with the Myc-associated transcriptional coactivators TRRAP/p434 and the histone acetyltransferase TIP60 and the stabilization of HTLV-1 p30^II^/Myc-TIP60 chromatin-remodeling complexes (73). Indeed, the retroviral p30^II^ accessory protein is recruited on the E-box enhancer elements within the endogenous cyclin D2 promoter along with c-Myc and the acetyltransferases TIP60 and p300, where lysine-acetylation of the c-Myc oncoprotein contributes to HTLV-1-induced carcinogenesis (79). Hence, in the context of HTLV-1 infection, Tax expression, in addition to p30^II^, appear to play a crucial role in polyploidy induction.

#### Hepatitis B Virus (HBV)

Hepatitis B virus (HBV), the prototype member of the *Hepadnaviridae* family (80), is an enveloped partly double-stranded DNA virus with characteristics similar to retroviruses, where reverse transcriptase is employed in the replication cycle (81). Hepatocytes are the major target and the confirmed site of HBV replication as the latter is identified as a hepatotropic virus, by virtue of controversial data on HBV detection in bile ductular epithelium and other extrahepatic sites (82). Viral replication in liver can precipitate transient and chronic infections with complications ranging from acute hepatitis to liver failure with cirrhosis and hepatocellular carcinoma (HCC) (83). Although endoreduplication and hyperploidy were identified exclusively in peripheral blood cells isolated from HBV chronic carriers compared the HBV negative individuals (84), the correlation between polyploidy and oncogenesis is distinct in the context of HBV infection. As a matter of fact, hepatocytes undergo progressive polyploidization, a postnatal process examined as an indicator of terminal differentiation and cellular senescence (85). A converse interrelationship exists between HCC and cellular polyploidy as evidenced by the increased number of diploid mononucleated hepatocytes capacities during HCC pathogenesis, thus setting the stage for rapid proliferation with higher mutagenic risk (86). Despite this, it is noted that in HCC, nuclear ploidy is positively correlated with poor prognosis and HBV infection where mononuclear polyploid fractions are increased significantly in HCC related to HBV infection (87). In this perspective, the hepatitis B virus X protein (HBx, also denoted as pX) is a viral protein with pleiotropic biological functions indispensable for viral replication (88). Indeed, HBx promotes cell cycle progression, inhibits the expression of tumor suppressor genes, for instance p53, and modulates methyltransferases transcription, alongside multiple functions that underline its potential role in HCC pathogenesis and cellular transformation (89). It has been shown that full-length expression of HBx in transgenic mice with liver fibrosis resulted in aberrant hepatocytes ploidy (90). Further, in biopsies of hepatocellular carcinoma patients, although the percentage of binucleated hepatocytes was reduced, a significant increase in DNA polyploidy was confirmed in HCC patients with actual or previous HBV infection (91). Interestingly, the impact of the full-length HBx (FL-HBx) expression is divergent in the setting of normal liver maturation, proliferation, and in liver carcinogenesis. Whereas, FL-HBx delays hepatocytes binucleation during maturation, a greater percentage of population enriched with polyploid cell with a significantly higher fraction of ≥8n nuclei is detected in HBV-infected mice. Most importantly, FL-HBx transgenic mice exhibited an important increase of 4n hepatocytes during HCC initiation, along with an increase in HCC progenitor cell markers (92). This is endorsed by the fact that in the HBV-positive hepatoma cell lines HepG2.2.15 and 1.3ES2, a higher hyperploidy is detected compared to HBV-negative cells (93). Although a high fraction of pX-induced polyploid cells undergo apoptosis in poorly differentiated, immortalized hepatocytes, the surviving cells demonstrated two-fold increase in polyploidy, displayed characteristics of oncogenic transformation and an increase in the expression of proliferation genes know to be elevated in HCC, an effect repressed in the absence of pX (94). In line with this, pX expression in immortalized mouse hepatocytes also induced polyploidy with a 2.5-fold increase in cells containing >4n DNA and in the nuclear size (95), as well as the formation of multinucleated cells (96). In parallel to HBx, HBV large surface protein (LHBs), one of the three HBV viral surface proteins encoded by preS1, preS2, and S open reading frame (ORF) is identified to exhibit oncogenic properties that can potentially contribute to hepatocarcinogenesis, as well as the mutated/truncated pre-S2 from (97). LHBs expression in the immortalized hepatic progenitor cell line NeHepLxHT demonstrated a self-propagating cycles of hyperploidy, which accounted for intra-tumor heterogeneity (93). Pre-S2 mutant LHBs-expressing hepatocyte-derived carcinoma HUH7 cells also showed a significant number of multinucleated cells (98). Furthermore, a positive correlation between LHBs, hyperploidy and increased nuclear size was detected in hepatocytes of patients with chronic hepatitis B (93). Taken together, HBx appears to be a key player linking HBV and polyploidy.

#### Hepatitis C Virus (HCV)

Hepatitis C virus (HCV) is a small single-stranded enveloped RNA virus that belongs to the family Flaviviridae (99). With narrow host specificity and tissue tropism, HCV is mainly hepatotropic, although several cell types are suggested to support viral replication, for example dendritic and epithelial cells, lymph nodes and others (100). Persistence of HCV infection paves the way to cirrhosis, severe liver disease and eventually HCC (101), in addition to a strong potential correlation with non-Hodgkin's B-cell lymphoma (102). In this setting, polyploidy has been also described (103). Peripheral blood mononuclear cells (PBMCs) isolated from HCV-infected patients presented chromosomal polyploidy compared to PBMCs of healthy individuals (6). Furthermore, a higher polyploidy frequency is detected in human and mouse primary hepatocytes infected by the HCV strain JFH-1 with respect to non-infected cells (104). Additionally, expression of the viral core protein, previously shown to induce HCC in transgenic mice (105), induced an extensive polyploidy in human liver cell line HepG2, and embryonic kidney cell line HEK293. In line with this, primary splenocytes, hepatocytes, and embryo fibroblasts isolated from core protein-expressing transgenic mice displayed almost two-fold polyploidy increase relative to control mice (104).

#### Polyomaviruses (PyVs)

Polyomaviruses (PyVs) are a family of small, icosahedral, non-enveloped, double-stranded DNA viruses. Advances in DNA sequencing technologies allowed the identification of various human PyVs including Merkel cell polyomavirus (MCPyV), John Cunningham Polyomavirus (JCPyV) and BK polyomavirus (BKPyV) (106) in addition to simian virus 40 (SV40), an oncogenic prototypical primate closely related to JCPyV and BKPyV (107). Although strong evidence suggest an etiological role of JCPyV and BKPyV in multiple human malignancies, only MCPyV is recognized as an oncogenic virus by being the causative agent of a rare aggressive skin cancer known as Merkel cell carcinoma (MCC) (108). Expression of MCPyV small T (sT) antigen, an oncoprotein crucial for viral replication with transforming capacities *in vitro* (109) resulted in the formation of multinucleated cells along with an increase in the >4n population in WI38 human diploid fibroblastic cells (110). sT induction in C57BL/6 mice resulted in the formation of poorly-differentiated neoplasia pleomorphic nuclei varying in size and shape in conjunction with multiple nucleoli (111). In agreement with this, BKPyV infection of renal proximal tubule epithelial cells resulted in the accumulation of polyploid cells (112), an effect also seen upon large tumor antigen (TAg) expression (113). Alternatively, JCPyV infection of both cerebral hemispheres revealed giant multinucleated astrocytes with pyramidal neurons harboring enlarged nuclei (114). Transfection with two JCPyV strains, namely Mad-1 and−98 resulted in an increased ploidy in RKO cells, a human colon carcinoma-derived cell line (115). In line with this, astrocytes infection with Mad-1 JCPyV resulted in the appearance of a tetraploid population, which expanded with the time of infection (116). JCPyV-infected atypical astrocytes were deemed to be in the hypertetraploid range (117). Further, JCPyV-inoculated fetal brain cell cultures showed a significant upsurge in polyploid cells with relatively elevated level of endoreduplication, an effect shared with the simian papovavirus SV40 (118). Indeed, SV40 infection of primary and secondary hamster embryo cells and primary mouse cells induced the formation of tetraploid cells, in addition to cells with a higher ploidy, ranging from 16n and 32n to 64n (119). In line with this, SV40-infected monkey kidney cells CV-1 ensued the detection of a fraction of cells with a DNA content in the tetraploid–polyploid (10–12n) range (120). Distinctively, SV40 large T antigen (LT), a multifunctional key protein in driving viral replication and cellular transformation induction, plays a crucial role in SV40-induced polyploidy (121, 122). CV-1 cells infected with a temperature-sensitive mutant T antigen developed tetraploidy at 37°C but not at 40.5°C, which highlights the T antigen function in polyploidy induction (123). A shift from diploid to tetraploid and octaploid population in human fibroblast strain IMR9O infected with SV40 was positively correlated with a concomitant increase in number of T antigen-positive cells (124). Furthermore, expression of LT alone induced tetraploidy in the hTERT-immortalized foreskin fibroblast cell line BJ/tert (125) as well as the formation of an 8n population in IMR90 and BJ cell lines (126). To note that polyploidy induction in human diploid fibroblasts and embryonic kidney cells was correlated with the expression of the SV40 large T antigen but not with the small T antigen (127).

In this context, it should be noted that the presence of giant polyploid cells following infection with non-oncogenic viruses is also described in the literature (128). However, distinction in various aspects compared to oncogenic viruses is prominent. First, induction of the polyploid phenotype is not coupled to transformation (129). Second, cell-cell fusion due to the presence of viral fusion proteins that lead to syncytia formation is the main mechanism of polyploidy induction in non-oncogenic viruses (130, 131), which is in contrast to the oncogenic viruses-employed molecular mechanisms discussed in the succeeding section.

### Several Biological Mechanisms Lead to PGCC Appearance in Oncoviruses Infections

**Figure 1:**
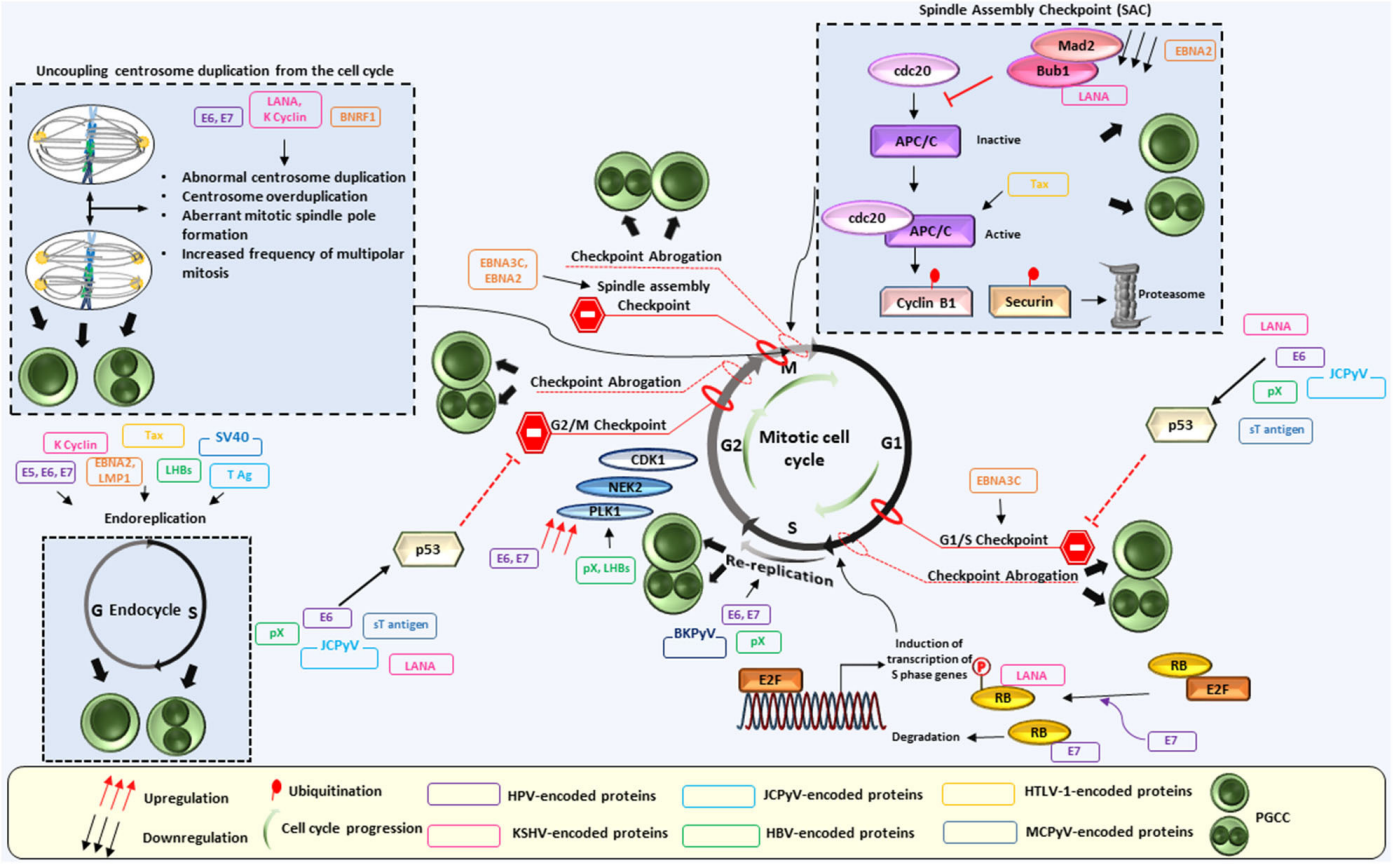
Interplay between oncoviral proteins and the cell cycle regulation leading to PGCC formation. Polyploidy has been described in the context of oncogenic viruses. Indeed, oncoviral proteins were shown to employ multiple cellular mechanisms to induce polyploidy. Those include centrosome overduplication and aberrant mitotic spindle pole formation, as well as abortive mitosis and cell cycle disruptions, The latter being dependent partly on p53 depletion or functional abolition, Rb inactivation or even through p53-independent mechanisms. Not limited to checkpoints abrogation, dysregulation of cell-cycle regulators activities such as polo-like kinase 1 (Plk1) also favor polyploidy induction, in addition to endoreplication and re-replication. Anaphase-promoting complex (APC), budding uninhibited by benzimidazoles 1 (Bub1), cell-division cycle protein 20 (cdc20), cyclin dependent kinase 1 (Cdk1), EBV nuclear antigen (EBNA), large HBV surface protein (LHBs), latency-associated nuclear antigen (LANA), latent membrane protein 1 (LMP1), mitotic arrest deficient 2 (Mad 2), NIMA-related protein kinase 2 (Nek2).

Multiple cellular mechanisms are employed by oncoviruses to induce polyploidy. Several models have been suggested, ranging from spindle assembly and postmitotic checkpoint abrogation, re-replication and hypoxia, to centrosome duplication, inactivation of tumor suppressors, Myc activation and others. Since those mechanisms can result in the inhibition of some or all aspects of mitosis, this may enhance the occurrence of abortive cell cycle, or alternatively endoreplication, one of the most described mechanisms of PGCC formation (6). In the following section, biological mechanisms mobilized or activated by oncoviruses in parallel to polyploidy induction are discussed (Figure 1).

#### Aberrant Centrosome Numbers

Centrosome is a vital microtubule organizing center with multiple functional complexities. Each cell contains one centrosome or alternatively two centrosomes that support the formation of a bipolar mitotic spindle during mitosis, thus ensuring accurate chromosome segregation (132). Centrosome abnormalities can induce the formation of additional mitotic spindle poles and subsequently genetic instability (133). HPV-16 E7 oncoprotein demonstrated its ability to induce abnormal centrosome duplication and aberrant mitotic spindle pole formation, which consequently uncoupled centrosome duplication from the cell cycle division and triggered genomic instability (134). This effect was potentiated by HPV-16 E6 and reserved to high risk HPV-16 (134), although independent expression of E6 and E7 was also demonstrated to induce centrosome amplification (135). On the other hand, accumulation of abnormal centrosome number in E6-expressing cells was positively correlated with multinucleated cells characterized by nuclear enlargement and more than or equal to 3 nuclei/cell, with a cellular subpopulation ultimately enduring replicative senescence (136). Polyploid cells with single large nucleus were linked to the expression of the EBV major tegument protein BNRF1, capable of inducing centrosome overduplication and increasing the frequency of multipolar mitosis (42). In line with this, KSHV infection induced multipolar and monopolar spindles along abnormal centrosome duplication, resulting in multinucleated cells with enlarged nuclei (53). This effect could be linked to the KSHV-encoded latent protein LANA, whose expression alone increased abnormal centrosome activities as well as the number of centrosomes to more than three per cell (56), in addition to KSHV K cyclin, in turn linked to the presence of 3–40 centrosomes per cell (61). Beside aberrant centrosome numbers, Aurora kinase B (AURKB) has been described to play an important role during mitosis as a regulator of chromosome segregation and cytokinesis (137), where it localizes with the centrosome during early mitosis and acts as a component of the chromosome passenger complex (138). Interestingly, KSHV latently infected tumor cells showed serine protease-N terminus cleavage of AURKB, an N'-cleaved form of AURKB protein also shared in malignant tumor cells induced by other oncoviruses including EBV and HPV. This proteolytic cleavage might promote a transition from metaphase to telophase, thus promoting mitotic progress and tumorigenesis (139). In this specific context, KSHV-encoded LANA upregulates Aurora A kinase transcriptional expression through targeting the Sp1 cis-element within the promoter, and in turn, Aurora A expression was shown to be critical for LANA-induced p53 ubiquitylation and degradation (140).

#### Abortive Mitosis and Cell Cycle Disruptions

Described as “guardian of the genome,” p53 responds to endogenous and exogenous genotoxic attacks and DNA-damaging agents by inducing a cell cycle arrest at the G1/S checkpoint (141). Subsequently, loss of p53 expression or activity prompts a failure to induce a G1/S transition arrest and predisposes cells to genomic instability (142). Beside the cell cycle, p53 exerts a G1-like growth arrest upon mitotic spindle damage to prevent DNA re-replication and polyploidy formation (143). For instance, p53-deficient cells were shown to from tetraploid and octaploid cells (4) and overexpression of mutant-type p53 enriched the formation of giant myelomonocytic leukemic M1/2 cells that further accumulated after gamma-irradiation (144), suggesting that loss of wild-type p53 can accommodate for the emergence of PGCCs (145). In the context of oncogenic viruses, p53 depletion engendered a five-fold increase of polyploid cells in response to HBV pX expression, demonstrating that p53 antagonizes pX-induced polyploidy (95). Besides, p53 loss coupled to Merkel cell polyomavirus sT antigen expression induced poorly differentiated tumors characterized by pleomorphic nuclei with considerable disparities in size and shape along multiple nucleoli and hyperchromatism, associated with stimulation of progenitor cell proliferation (111). Polyploidy detected in the context of JCPyV infection was also correlated with functional abolition of p53 (117). On the other hand, HPV E6 oncoprotein was shown to interfere and inhibit all the p53-mediated functions (146). As a consequence, HPV16 E6-expressing cells abrogated the mitotic checkpoint, resulting in the loss of G2/M arrest and the appearance of a substantial 8n population (147). Interestingly, E7-expressing cells were also demonstrated to overcome the mitotic checkpoint by a p53-independent mechanism, possibly by deregulating Rb function and overexpressing Mdm2 (147), a cellular oncogene capable of inducing several rounds of S-phase replication without transition through mitosis (148). On the other hand, concomitant E6 and E7 expression induced polyploidy due to disruption of the spindle checkpoint and up-regulation of the G2-M proteins including Aurora-A, Nek2, cyclin-dependent kinase 1 (Cdk1) and polo-like kinase 1 (Plk1), the latter being dependent on p53 degradation and repression of pRb functions mediated by E6 and E7, respectively (149). Not limited to the abrogation of the spindle checkpoint, a p53-independent mechanism of E6-induced abrogation of the postmitotic G1-like checkpoint after adaptation of the mitotic stress is evidenced by the fact that E6 mutants defective in p53 degradation were also capable of inducing polyploidy (27). This could be elucidated by the reduced nuclear p21 localization and the active expression of Cdk1 that is mediated by E6-upreglated E2F1 upon microtubule disruption (28). This postmitotic checkpoint abrogation-induced polyploidy is also shared by E7 through its Rb-downregulation function (32). On the other hand, KSHV-encoded LANA was shown to deregulate both p53 and Rb pathways (150) and its expression can induce S-phase entry by protecting from the cell cycle arrest induced by the CDK4/6 inhibitor p16 INK4a (151). Subsequently, LANA-expressing cells showed multinucleation, abnormal spindle pole activities and an increased entry into S phase through LANA-mediated suppression of p53 transcription and transactivation activity, partly by repressing its endogenous promoter (56). In addition, by directly functioning as a component of the EC5S ubiquitin complex and interacting with the Cul5-Elongin BC complex, LANA expression in KSHV-infected B lymphoma cells induces polyubiquitylation and subsequently degradation of p53 and von Hippel–Lindau (VHL), another tumor suppressor (152). On the other hand, expression of EBV latent gene compromises the mitotic spindle assembly checkpoint and consequently averts metaphase arrest (149). Further, expression of EBNA3C, a protein with oncogenic activity comparable to E7 (46), induced cell cycle progression without cytokinesis in the absence of external mitogenic signals and not only deregulated the G1 checkpoint, but also disrupted the spindle assembly checkpoint, resulting in polyploidy (47), the latter effect also shared by EBNA2, another EBV protein with oncogenic activities (45). In addition, a prominent role of EBNA3C is pointed in this context as it can interact and transcriptionally regulate a wide array of cellular and viral transcriptional factors (153). For instance, EBNA3C N-terminal region was shown to complex with the N-terminal pRb binding domain and C-terminal domain of cyclin D1, which not only stabilizes cyclin D1 through the inhibition of its ubiquitin-mediated proteasomal degradation, but also enhances the functional kinase activity of cyclin D1/CDK6, thus facilitating the G1-S transition (154). Repression of p53 transcriptional activity and sparing p53-induced apoptosis in osteosarcoma cells was mediated by EBNA3C direct interaction of the latter N-terminal domain with p53 C-terminal DNA-binding and tetramerization domain (155). Also, EBNA3C directly interacts with the RNA helicase Gemin3, which stabilizes the latter and promotes formation of a complex with p53, subsequently blocking the DNA-binding affinity of p53 and p53-mediated transcriptional activity and apoptosis pathway (156). EBNA3C physically interacts with the oncoprotein Pim-1, enhancing Thr145 phosphorylation and proteosomal degradation of p21, thus enhancing cellular proliferation (157). Hence, a wide variety of oncoviruses-induced defects in distinct aspects of cell cycle regulation and checkpoints can give rise to abortive cell cycle and polyploidy.

#### Dysregulation of Cell-Cycle Regulators Activities

Cell cycle is a highly regulated multipronged process orchestrated by a suite of cell-cycle regulators (158). Dysregulation of any of these regulatory mechanisms can result in genomic instability and polyploidy (159). EBNA2 was shown to specifically downregulate the mitotic arrest deficient 2 (MAD2) and upregulate Plk1, which can result in the activation of the anaphase promoting complex/cyclosome (APC/C) and subsequently securin degradation, thus engendering a metaphase-anaphase transition and opening the door toward polyploidy (45). In line with this, expression of the HBV LHBs upregulated Plk1, resulting in a Plk1-dependent G2/M checkpoint override (93). Not limited to LHBs, HBS pX protein mediated the activation of Plk1 in G_2_ phase (160). This attenuated the DNA damage checkpoint through induction of clapsin proteasomal degradation and increased the inhibitory Tyr15-Cdc2 phosphorylation, as well as the suppression of p53-mediated apoptosis, promoting polyploidy generation (92). In line with this, pX-induced upregulation of Plk1 through p38 and ERK1/2 pathways can induce an attenuation of cell cycle checkpoint control, leading to an increase in polyploidy, DNA damage propagation and hepatocyte transformation, beside increased markers of cancer progenitor cells, such as AFP, Ly6D, and EpCam (90, 92). Indeed, Plk1 is crucial in pX protein-mediated oncogenic transformation, including pX-induced polyploidy, as Plk1 inhibition suppresses pX oncogenic potential (94). On the other hand, HTLV-1 oncoprotein Tax drastically reduced Pds1p/securin and Clb2p/cyclin B levels through the activation of the Cdc20p-associated anaphase-promoting complex APCCdc20p (161). Furthermore, Tax was shown to directly bind to TXBP181, a homolog of mitotic check-point MAD1 protein and target its mitotic checkpoint function by reducing its dimerization and stability, hence leading to the loss of the M checkpoint and the appearance of multinucleated cells (69). On the other hand, a key protein component of the spindle checkpoint, Bub1, was shown to interact with the KSHV-encoded LANA, which in turn promotes Bub1 ubiquitination and degradation via the APC/C E3-Ub ligase, thus favoring multinucleation (57).

#### Endoreplication

In contrast to the mitotic cycle that ensues the maintenance and the distribution of the same amount of genetic content between mother and daughter cells, endoreplication, or endoreduplication cycle consists only of G and S phases with no features of mitosis, a process termed endocycle, beside endomitosis, an abortive mitosis without cell division. This generates polyploidy with cells containing separate multiple nuclei or an enlarged, single nucleus harboring all the genetic content (162). Indeed, Zhang et al. reported endoreduplication as a potential mechanism of PGCC formation after cobalt chloride treatment (163). HPV16 E5 is capable of inducing endoreplication (34), a mechanism shared by E6 oncoprotein as evidenced by the presence of intracellular cytokinesis (164). Transduction with HPV-18 E7 pushed the cells to reenter another round of S phase resulting in two consecutive S phases without cytokinesis, hence ensuing multinucleated cells with enlarged nuclei (35). In addition, re-entering S phase and synthesizing DNA was also triggered in EBNA2-expressing cells (45) and LMP1 expression (49). Besides, DNA replication and cell nuclei division without concomitant cell division yielded large or multiple nuclei upon KSHV K cyclin expression (61). HTLV-1 Tax oncoprotein uncoupled DNA synthesis from cell division by activating G1/S entry and blocking mitosis, inducing the formation of multinucleated giant cells (161). Furthermore, cells expressing HBV LHBs demonstrated cytokinesis failure compared to controls (93). JCPyV T antigen was similarly shown to induce endoreplication, resulting in an excess of polyploidy (118). Endoreplication is also shared by SV40 with synchronous progression through the cell cycle into the first S phase, followed by a second S phase (120), an outcome dependent on SV40 large T antigen expression (121). This could be explained by an unappreciated *in vivo* interaction between large T antigen and nibrin (Nbs1), resulting in the formation of a T/Nbs1 complex that interferes with Nbs1 role in suppressing DNA replication re-initiation (126). Nonetheless, T antigen-induced tetraploidy was also shown to be dependent on the binding of the latter to the mitotic checkpoint serine/threonine kinase Bub1 (125).

#### Re-replication

In contrast to endoreplication, re-replication is a successive rounds of DNA synthesis within a given S phase without entering mitosis, leading consecutively to polyploidy (165). Polyploidy formation in HPV-16 E7-expressing cells was mediated through re-replication in response to DNA damage after being arrested at G2 checkpoint, as evidenced by the upregulation of the DNA replication initiation factor Cdt1, a gene responsible of triggering re-replication upon overexpression (29, 166). Another player in E7-induced re-replication is the DNA replication initiation factor known as the cell division cycle 6 (Cdc6) whose downregulation reduces E7-induced re-replication (30). Indeed, Cdc6 protein is expressed in both cervical squamous carcinoma and adenocarcinoma and is upregulated possibly due to E7-mediated release of E2F inhibition upon Rb binding, since Cdc6 is an E2F responsive gene (167). In addition, HBV pX-induced polyploid cells were shown to undergo DNA re-replication, beside aberrant mitotic spindles (94). Indeed, pX-mediated DNA re-replication was correlated with Cdt1 upregulation and Cdc6 expression, both required for pre-replicative complex assembly, along with suppressed expression of geminin, the main inhibitor of replication licensing in S and G2 phases (95). BKPyV was also demonstrated to induce multiple rounds of DNA replication within a single cell (112). This could be explained by a synergistic cooperation between ataxia telangiectasia, mutated (ATM) and ataxia telangiectasia, and Rad3-related (ATR), where ATM promotes efficient S-phase entry and mitosis block after complete DNA replication, and ATR arrests the cell cycle to prevent entry into mitosis in actively replicating cells, resulting in BKPyV-mediated polyploidization (113).

#### Hypoxia

Hypoxia is identified as a critical microenvironmental factor involved in tumor progression as well as maintenance and self-renewal of cancer stem cells. This is arbitrated by the hypoxia inducible factors (HIFs) and mediated through various mechanisms, including but not limited to activity enhancement of stem cell factors such as OCT-4, c-Myc, and Nanog (168). Hypoxia mimic was shown to underlie the formation of PGCC, the latter then contributing to the generation of cancer stem-like cells (163, 169). Notably, most oncogenic viruses are capable of deregulating cellular hypoxia-inducible factor 1 (HIF-1) signaling pathway and enhancing HIF-1 levels (170), the latter mediating a fundamental role in transcriptionally upregulating metabolic, angiogenic, and microenvironmental factors indispensable for oncogenesis (171). For instance, HPV-16 oncoproteins E6 and E7 significantly promote HIF-1a protein accumulation and enhanced activity in human cervical cancers (172). EBV LMP1, EBNA-1, EBNA-3, and EBNA-5 enhance the rate of HIF-1α synthesis, HIF-1α gene transcription and inhibit its breakdown, respectively (173–175). KSHV interferon regulatory factor 3 (vIRF-3) also called LANA-2 increases HIF-1α nuclear translocation and activity through direct binding (176). Mediated through several putative hypoxia response elements, LANA direct association with HIF-1α enhances HIF-1α mRNA level and transcriptional activities (177) and is compulsory for efficient viral replication in the hypoxic microenvironment through the inhibition of the cellular E3-ubiquitin ligase-mediated proteosomal degradation (178), as well as lytic and latent viral genome regulation through a unique SUMO-interacting motif during hypoxia (179). Furthermore, a KSHV-encoded G protein-coupled receptor (GPCR) stimulates HIF-1α through the phosphorylation of its regulatory/inhibitory domain, which enhances its transcriptional activity, resulting in transcriptional activation of the vascular endothelial growth factor (VEGF) promoter, a crucial angiogenic stimulator (180). This sheds the light on the potential therapeutic targeting of HIF-1α in the context of KSHV-induced PEL as HIF-1α activity is stimulated by KSHV infection and HIF-1α activates numerous KSHV genes. This is reinforced by the fact that HIF-1α suppression was associated with a significant inhibition of PEL growth and a reduction in the activation of KSHV lytic and latent genes (181). In addition, HBV HBx interferes with HIF-1α protein degradation (182) and enhances its synthesis (183). HCV core protein upregulates HIF-1α mRNA transcription (184), whereas HTLV1 Tax enhances its protein expression (185). Therefore, although employing divergent mechanisms, HIF-1 activation by enhancing its transcription, translation, or stabilization appears to be a common pathway among oncogenic viruses. Given that most of these studies outline the mechanisms underlying such activation and the subsequent downstream activation of key cancer-promoting genes, polyploidy assessment in the context of viral-induced HIF-1 activity is underestimated and future studies are undoubtedly indispensable to reveal the interrelationship between the triad of oncogenic virus, hypoxia and polyploidy.

#### Cellular Senescence

Typically considered in a senescent state, polyploid cells were generally examined as a tumor suppressor mechanism as senescence is characteristically considered a terminal cell fate (186). Nevertheless, PGCCs have been shown to escape senescence, generate daughter cells that could undergo mitotic cell divisions (187) and contribute to immortalization and transformation (188). In the context of latent KSHV infection, the viral v-cyclin protein was shown to induce senescence by deregulating the cell cycle and activating the DNA damage response (DDR) subsequent to aberrant host DNA replication. Interestingly, viral FLICE inhibitory protein (v-FLIP) can bypass senescence and facilitate the growth and division of latently infected cell population (189) that can comprise a subpopulation of polyploid cells as discussed previously. It is worthy to note that v-FLIP is present in several gamma-herpesviruses, including EBV, as well as in the tumorigenic human molluscipoxvirus (190). Hence, assessing the previously discussed v-FLIP function in the context of polyploidy induction could be of interest. On the other hand, HTLV-1 Tax induced senescence, accompanied by cells with enlarged nuclei or two nuclei, with a special emphasis on a small subpopulation that was found to circumvent Tax-induced rapid cellular senescence (70).

#### Telomere Dysfunction

As telomeres are essential for preserving chromosome stability, progressive telomere dysfunction triggers cytokinesis failure and engenders polyploidy (191). Expression of LMP1, the primary transforming gene product of EBV led to a significant increase in telomeric aggregates and a decrease of the total telomere number, with a significant increase of the number of multinucleated cells, as well as the nuclear volume. This perturbation of telomere protection was correlated with a suppression of shelterin proteins TRF1, TRF2, and POT1 responsible of preserving the telomere structure and its signaling functions (49, 192). In particular, TRF2 displacement from telomeres in EBV-infected cells was shown to significantly contribute to genomic instability (193).

Overall, oncoviruses deploy multiple mechanisms that could be regarded as precursor of polyploidy and driver of oncovirus-induced human cancers, tumor progression, and intratumoral heterogeneity. Interestingly, human cytomegalovirus (HCMV), a beta herpes virus with high oncogenic potential (194) shares most of the previously mentioned mechanisms of polyploidy induction. For instance, CMV infection not only induced supernumerary centrosomes, but also formation of abnormal mitotic spindles (195). In addition, several HCMV proteins were shown to interact with p53 (196). As an illustration, immediate early 2 protein demonstrated its ability to interact with p53 *in vitro* and *in vivo*, transcriptionally inactivating the latter (197). Besides, HCMV UL97 inactivates Rb by phosphorylation, whereas pp71 binds to the Rb family proteins and induces their proteasomal degradation (198). HCMV was also shown to activate Myc at the transcriptional level (199) as well as at the translational level (200) and to induce HIF-1α expression (201). In addition, multinucleated giant cells formed by cell fusion were detected following HCMV infection (202) as well as an up-modulation of the expression levels of the G_2_/M transition regulators, including the previously mentioned Plk1 (195). This emphasizes the fact that primary pathways and mechanisms of polyploid formation could be shared among oncogenic and potentially oncogenic viruses, and points toward the potential elaboration of an empirical hypothesis linking those viruses to polyploid induction and tumorigenesis.

### New Therapeutic Approaches Are Actively Needed to Fight PGCC, but Also Oncoviruses

Recently, a growing number of evidence pointing toward polypoid giant cancer cells as key actuators of therapy resistance, metastasis, and relapse is emerging (203). For instance, PGCC purified from ovarian cancer cells were more resistant to treatment with cisplatin (163). Furthermore, giant cells selected from high metastatic human prostate cancer not only showed resistance to 5-fluorouracil (5-FU), doxorubicin and cisplatin, but also potently developed metastasis in lung, bone, and some major lymph nodes, including popliteal, inguinal, axillary, and cervical nodes (204). Large multinucleated cells in murine fibrosarcoma were more resistant to doxorubucin and established orthotopic subcutaneous tumors with spontaneous lung metastases (205). In addition, human colon cancer cells exposed to cycles of 5-FU, oxaliplatin, and irinotecan treatment to mimic the clinical therapeutic regimens demonstrated a resumption of proliferation and cancer re-population, a finding principally attributed to the progeny of the established polyploid giant cells (206). In the context of oncogenic viruses, although an explicit link with therapy resistance and PGCC is lacking, some interpretations could be drawn (Table 1).

**Table 1:** Anti-tumor treatment, PGCC, and oncoviruses.

**Viral agent**	**Oncoproteins involved**	**Cancer cells lines/tumor types**	**Type of therapy resistance acquired**	**Associated/described outcomes**	**References**
Epstein–Barr virus (EBV)	Latent membrane protein 1 (LMP1) Epstein–Barr nuclear antigen 2 (EBNA2) Epstein–Barr nuclear antigen 3C (EBNA3C) BNRF1	Burkitt's lymphoma-derived cells	Radioresistance	Appearance of cells with grossly abnormal and multiple nuclei	(149)
			Chemoresistance (taxol)	Appearance of polyploid cells with aberrant nuclei	(149)
		BHRF1-expressing nasopharyngeal carcinoma cells	^60^Co radioresistance	Increase in colony formation ability, cell proliferative rate and tumor formation in nude mice	(207)
		LMP1-expressing nasopharyngeal carcinoma cells	Radioresistance	Induction of proliferation, invasion and apoptosis suppression	(208)
Human papillomavirus (HPV)	E5 E6 E7	HPV-positive cervical cancer cells CaSki	Chemoresistance (camptothecin and cisplatin)	Continued proliferation to confluency and lack of apoptotic features despite prolonged exposure to chemotherapeutic agents	(209)
		HPV16 E6 and E7-expressing esophageal squamous cell carcinoma	Chemoresistance and radioresistance	Reduction in G0/G1 cell cycle arrest, establishment of a cancer stem-like phenotype, anti-apoptotic effect and enhancement of migration, invasion and spherogenesis	(210)
		HPV E6-positive cervical cancer cells	Radioresistance	Enhancement of self-renewal ability, proliferation and tumorigenicity, and increase in transcriptional levels of some genes related to stemness (e.g., OCT-4, Nanog, ABCG2 and Bmi-1)	(211, 212)
		HPV16-positive cervical cancer cell line SiHa	Chemoresistance (5-fluorouracil)	Enhancement of migration and invasion potentials with elevated levels of functional and molecular markers of epithelial-to-mesenchymal transition (EMT) (e.g., Snail, Slug, Twist, vimentin)	(213)
Hepatitis B virus (HBV)	Hepatitis B virus X protein (HBx) HBV large surface protein (LHBs)	pX-transfected hepatocellular carcinoma cells	Chemoresistance (pirarubicin, oxaliplatin and hydroxycamptothecin)	Increase in tumorigenecity, self-renewal and stemness-associated genes expression (e.g., CD133, Nanog, SOX-2 and OCT-4)	(214)
Kaposi's sarcoma herpes virus (KSHV)	Latency-associated nuclear antigen (LANA) Cyclin K	LANA-expressing human breast cancer cell line MCF-7	Chemoresistance (paclitaxel)	Inhibition of G_2_ arrest and microtubule polymerization in response to paclitaxel	(215)
Human T-lymphotropic virus type 1 (HTLV-1)	Tax	Cells derived from adult T-cell leukemia/lymphoma patients or HTLV-1 transformed cells (MT-2 and MT-4)	Chemoresistance (etoposide- and doxorubicin)	Elevated expression levels of the antiapoptotic proteins	(185)

#### A Link Between Oncoviruses, PGCC and Therapy Resistance Is Observed in Several Cancers

It has been shown that EBV expression rescued Burkitt's lymphoma-derived cells from death after gamma-irradiation and resulted in the appearance of recovered cells with grossly abnormal and multiple nuclei, compared to rapid death in EBV-negative cells (149). Similar results were also reported at 72 h post-treatment with taxol, where treated cells became polyploid and developed aberrant nuclei in the EBV-positive population, versus a mass cellular death in EBV-negative controls (149). Further, nasopharyngeal carcinoma cells expressing EBV gene BHRF1 recovered faster after ^60^Co radiation with higher cell proliferative rate and colony formation ability, beside a greater tumor formation in nude mice compared to control groups (207). This is in line with the EBV LMP1-induced proliferation, invasion, apoptosis suppression, and radioresistance in NPC cells (208).

HPV-positive cervical cancer cells exhibit an inherent chemoresistance to both camptothecin and cisplatin (209). HPV16 E6 and E7 reduced G0/G1 cell cycle arrest, promoted a cancer stem-like phenotype and an anti-apoptotic effect, enhanced migration, invasion, spherogenesis, and increased chemoresistance and radioresistance after ionizing radiation in esophageal squamous cell carcinoma (210). A subpopulation of HPV-E6 positive cervical cancer cells enriched with CD71, a glycoprotein detected with poorly differentiated acute myeloid leukemia (216) and radioresistant glioma cells with cancer stem-like cells properties (217), demonstrated resistance to irradiation, an enhanced self-renewal ability, proliferation and tumorigenicity (211), in addition to higher transcriptional levels of some genes related to stemness including OCT-4, Nanog, ABCG2, and Bmi-1 (212). Furthermore, HPV16 E6 was shown to be actively involved in migration and invasion potentials, with elevated levels of functional and molecular markers of epithelial-to-mesenchymal transition (EMT) such as Snail, Slug, Twist, and vimentin, which could promote chemoresistance in cervical cancer (213).

Interestingly, hepatocellular carcinoma cells transfection with HBV pX protein contributes to the expansion of a subpopulation within the total HCC cell pool characterized by an increased tumorigenecity, self-renewal, stemness-associated genes expression such as CD133, Nanog, SOX-2, and OCT-4, and an enriched chemoresistance toward pirarubicin, oxaliplatin, and hydroxycamptothecin (214). This property is correspondingly shared in the setting of EBV-associated NPC (218).

KSHV latent protein LANA2 induces paclitaxel resistance, which suggests a potential correlation between LANA2 expression and the resistance to paclitaxel in the setting of primary effusion lymphoma (215).

HTLV-1-infected cells derived from adult T-cell leukemia/lymphoma patients or the HTLV-1 transformed cells MT-2 and MT-4 were highly resistant to etoposide and doxorubicin with elevated expression levels of the anti-apoptotic proteins mediated by Tax (185).

It is remarkable that many of the discussed tumors fall under the category of carcinoma or adenocarcinoma, where epithelial or epithelial-like cells are identified as a major system where PGCCs develop. Indeed, the latter are frequently described in similar settings, for instance prostate (219), colon (220) and breast cell lines (221), in addition to ovarian tumors (222), which could suggest that the polyploid phenotype could be interrelated to the origin of the cell or favored by the microenvironment.

#### Highlighting a Biological Model Where Oncoviruses, Through the Generation of PGCC, Could Favor the Establishment of Therapy Resistance, Metastasis and Relapse

**Figure 2:**
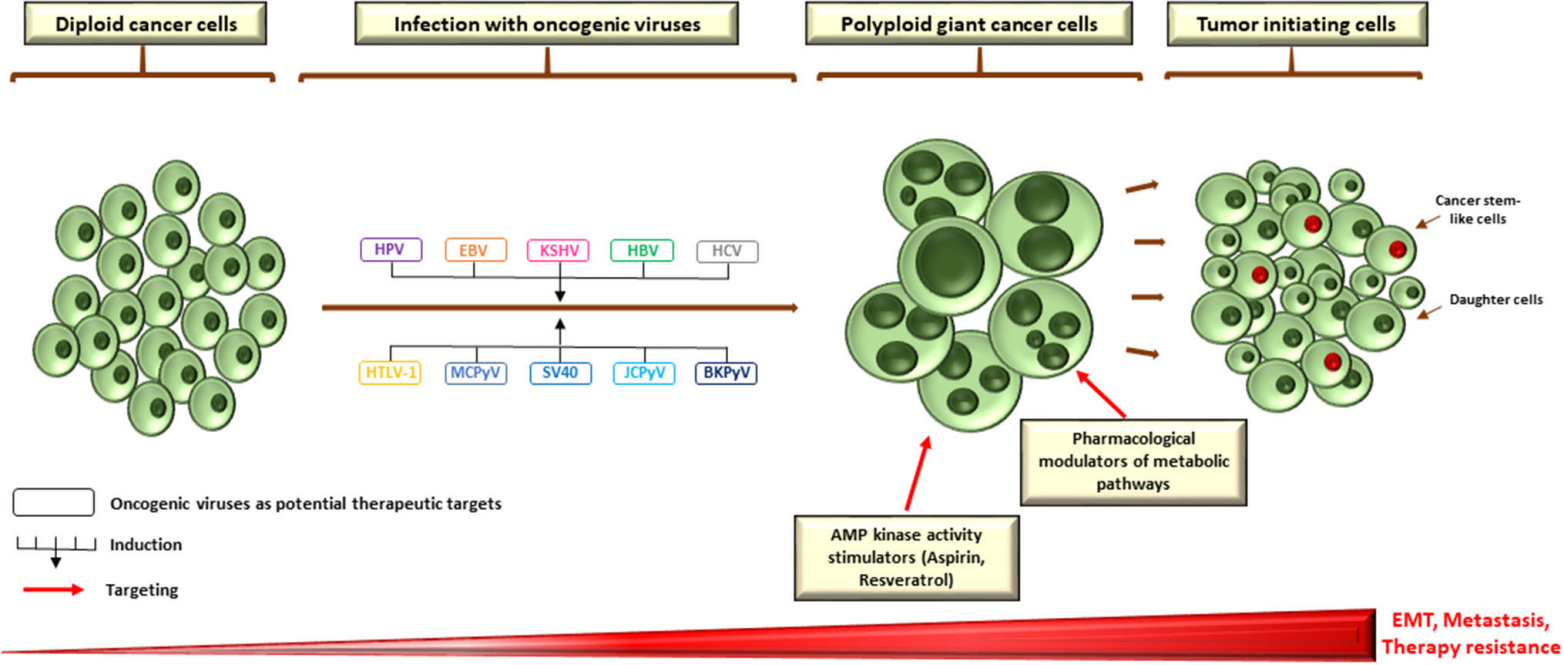
Potential therapeutic targets to block PGCC formation during infection with oncoviruses. As a growing body of evidence is pointing toward a crucial role of polypoid giant cancer cells in enhancing tumor evolution and the acquisition of therapy resistance, therapeutically targeting those cells is of utmost importance. Pharmacological modulators of metabolic pathways and AMP kinase activity stimulators appear to be promising candidates that specifically target polyploid tumor cells to counteract tumor repopulation. However, as oncogenic viruses might be mediators and/or inducers of PGCC formation, targeting those pathogens could also constitute a potential therapeutic strategy to block the formation of giant cells, which could disrupt the adaptive capacity of tumors *in vivo* and establish an effective strategy in the war against cancer. EBV nuclear antigen (EBNA), EMT (epithelial-to-mesenchymal transition) large HBV surface protein (LHBs), latency-associated nuclear antigen (LANA), latent membrane protein 1 (LMP1).

Genuinely, a robust evidence relating polyploidy, cancer stem-cell phenotype and EMT do exist (3). For instance, upregulation of OCT4, SOX2, and Nanog in the setting of irradiated lymphoma cells was clearly detected in endopolyploid tumor cells, which can resist apoptosis, overcome cellular senescence and transfer this primitive phenotype to descendants through de-polyploidisation (223). Furthermore, reprogramming of differentiated breast cancer cells following ionizing radiation ensued only in a subpopulation of polyploid cells with re-expression of OCT-4, SOX-2, Nanog, and Klf4 (224). This is in line with expression of OCT-4, Nanog, SOX-2, and SSEA1 in PGCCs whose daughter cells demonstrate an increased resistance to paclitaxel (225). Interestingly, PGCCs and PGCC-derived daughter cells gained a mesenchymal phenotype as demonstrated by the enhanced expression of some markers such as Snail, Slug, Twist 2, vimentin, and others (163, 225). This not only indorses invasion and metastasis, but also may convey a drug refractory state due to therapy resistance (226). Taken together, it could be hypothesized that oncogenic viruses might, through the induction of polyploidy, play a central role in drug resistance, metastasis, and relapse of human cancer through the establishment of primitive cellular phenotypes. As the presence of such pathogens could compromise therapy, targeting those viruses with the purpose of blocking the formation of PGCC may potentially have some clinical implications as a promising anti-cancer therapeutic approach, in conjunction to the newly proposed therapeutic strategies to target polyploidy, for instance the pharmacological AMP kinase activity stimulators or modulators of metabolic pathways (Figure 2) (3, 227).

## Perspectives and Conclusion

Overall, synthesizing the diverse literature on polyploidy converges to argument that an interrelationship might exist between infection with oncogenic pathogens, the polyploid phenotype, and tumorigenesis. This is reinforced by the fact that chromosomal changes occurred at the time of morphological transformation (119) and that the multinucleated cell phenotype was induced upon the expression of viral oncoproteins, rather than by trivial possibilities such as cell fusion (68, 228), the latter being also shared by other non-oncogenic pathogens (128). Nonetheless, and despite the prevalence and importance of polyploidy, the assessment of such phenotype is underestimated in the setting of oncoviruses. Several questions in terms of polyploidy generation as well as maintenance remained unanswered. Those include not only the contribution of each mechanism and the potential cooperation between the various signaling pathways, but also the estimated rates of polyploidy formation, in addition to the mechanism of stemness profile acquisition that arms this population with the advantage to propagate, form a subclonal populations and convey therapy resistance, although underassessed in this context. Addressing those questions is further sophisticated by the presence of viral proteins that might confer an additional layer of complexity to the tumor microenvironment. Conceptualizing the hypothesis outlined in this review warrants a collective effort to link the described findings to polyploidization and transformation. This will provide an enhanced mechanistic understanding of some molecular mechanisms and pave the road toward a novel perception of effective and innovative therapeutic targets. Thus, new modalities of treatment are encouraged not only to disrupt the viral replicative machinery and to block the immortalizing and transforming capacity of oncogenic viruses, but also to target PGCC, with the ultimate goal of rendering polyploidy a “druggable” phenotype, with a special emphasis on disease relapse and therapy resistance.

## Author Contributions

ZN and GH wrote the article and draw the figures. All authors contributed to the article and approved the submitted version.

## Conflict of Interest

The authors declare that the research was conducted in the absence of any commercial or financial relationships that could be construed as a potential conflict of interest.

## Abbreviations

APC/C: anaphase promoting complex/cyclosome
ATL: adult T-cell leukemia/lymphoma
ATM, ataxia telangiectasia: mutated
ATR: ataxia telangiectasia and Rad3-related
AURKB: aurora kinase B
BL: Burkitt's lymphoma
Cdc6: cell division cycle 6
CDK: cyclin-dependent kinase
DDR: DNA damage response
EBNA: Epstein–Barr virus nuclear antigen
EMT: epithelial-to-mesenchymal transition
GPCR: G protein-coupled receptor
HCC: hepatocellular carcinoma
HHV8: human herpesvirus 8
HGSIL: high-grade squamous intraepithelial lesion
HR: high-risk
KS: Kaposi sarcoma
LANA: latency-associated nuclear antigen
LMP1: latent membrane protein 1
LR: low-risk
MAD2: mitotic arrest deficient 2
MCC: Merkel cell carcinoma
NPC: nasopharyngeal carcinoma
ORF: open reading frame
PBMCs: peripheral blood mononuclear cells
PGCC: polyploid giant cancer cells
Plk1: polo-like kinase
VEGF: vascular endothelial growth factor
VHL: Von Hippel-Lindau
v-FLIP: viral FLICE inhibitory protein.
